# The assessment of thyroid autoantibody levels in euthyroid patients with polycystic ovary syndrome

**DOI:** 10.4274/jtgga.2018.0001

**Published:** 2018-11-15

**Authors:** Sema Hepşen, Melia Karaköse, Erman Çakal, Sanem Öztekin, İlknur Ünsal, Pınar Akhanlı, Bekir Uçan, Mustafa Özbek

**Affiliations:** 1 Clinic of Endocrinology and Metabolism, University of Health Sciences, Dışkapı Yıldırım Beyazıt Training and Research Hospital, Ankara, Turkey; 2 Clinic of Endocrinology and Metabolism, Sivas Numune Hospital, Sivas, Turkey; 3 Clinic of Internal Medicine, University of Health Sciences, Dışkapı Yıldırım Beyazıt Training and Research Hospital, Ankara, Turkey

**Keywords:** Polycystic ovary syndrome, autoimmunity, anti-thyroid peroxidase antibody, anti-thyroglobulin antibody

## Abstract

**Objective:**

Thyroid hormone abnormalities are commonly seen in polycystic ovary syndrome (PCOS) and have considerable effects on comorbidities. The association with PCOS and thyroid autoimmunity which lead to thyroid pathologies are not revealed clearly. We targeted to commentate anti-thyroid peroxidase (anti-TPO), anti-thyroglobulin (anti-TG) antibody levels and thyroid autoimmunity in PCOS.

**Material and Methods:**

One hundred eighty four patients who got the diagnosis of PCOS regard to the revised 2003 Rotterdam criteria were embodied in this study. One hundred six age-matched female volunteers were included in the control group. Characteristics, biochemical parameters, thyroid hormone and autoantibody levels of groups were investigated.

**Results:**

Although; we did not find out a statistically significant difference in TSH and sT4 levels between two groups (p>0.05), anti-TPO and anti-TG antibody levels were determined higher in PCOS group significantly (p<0.001). Anti-TPO Ab and anti-TG Ab positivity prevalence of PCOS patients were significantly higher as against to controls (p<0.001; p=0.01).

**Conclusion:**

Not only thyroid hormone levels but also thyroid autoantibody levels should be screened during the investigation of PCOS and the patients with positive results need to be followed up carefully in the long run.

## Introduction

Polycystic ovary syndrome (PCOS) is a prevalent endocrinologic disorder affect women at the fertility period (1). PCOS is identified with menstrual irregularity, hyperandrogenism, and infertility (2). Obesity, metabolic syndrome, dyslipidemia, insuline resistance, type 2 diabetes mellitus, and cardiovascular disorders are the most common comorbidities related to this syndrome (3,4,5,6). 

Autoimmune thyroid disease prevalence in women is 4% and rises up to 15% in the event of existing thyroid autoantibody positivity (7). Thyroid hormone dysfunctions and thyroid autoimmunity cause abnormalities on sex hormone metabolism, menstrual irregularities and consequently infertility (8,9).

Anti-thyroid peroxidase antibodies (anti-TPO Ab) and anti-thyroglobulin antibodies (anti-TG Ab) are fundamental markers of thyroid autoimmunity. A study by Poppe et al. (8) demonstrated that thyroid auto antibodies are significantly higher in infertile patients. Close follow up of thyroid hormones are considered important in patients with PCOS because of being the most common reason of medically treatable infertility (10). The latest studies revealed that autoimmune thyroid diseases have an increased prevalence in PCOS patients (11,12). From this point of view, not only thyroid hormones are substantial for PCOS follow-up but also thyroid antibodies can be guiding for probable thyroid diseases.

The target of this study is to assess thyroid autoantibodies and thyroid hormone levels in PCOS patients by taking into consideration with present or probable thyroid hormone dysfunctions can affect patient’s clinical conditions and fertility substantially.

## Material and Methods

Our study includes 184 patients who got PCOS diagnose in regard to the revised 2003 Rotterdam criteria at the Endocrinology and Metabolism and Internal Medicine departments of our institution between January 2014-April 2015 (13). The definition criteria include at least two of the three following subheadings after exclusion of related disorders; oligo or anovulation, clinical and/or biochemical signs of hyperandrogenism and ultrasonographic demonstration of polycystic ovary appearance (13).

One hundred six age-matched healthy female volunteers who menstruate regularly were included in the study as the control group. Individuals who have the diagnosis as hyperprolactinemia, congenital adrenal hyperplasia, androgen-secreting tumours, Cushing syndrome, hypertension, hepatic or renal insufficiency, diabetes mellitus and concurrent thyroid dysfunction were excluded from the study. Being in pregnancy or breastfeeding period and using drugs which affect glucose tolerance and lipid levels were other exclusion criterias. The age range was between 18-41 for all participants. The study protocol was granted by the Ethics Department and each individual signed a written informed consent form. Clinical and anthropometric data including body mass index (BMI) and waist/hip ratio were ascertained for each participant.

Biochemical parameters of all individuals were studied after 12 hours fasting at 2^th^-5^th^ days of the follicular phase. Chemiluminescent immunoassay method was used to assess fasting blood glucose levels (Advia Centaur XP, Siemens Healthcare Diagnostic Inc., Tarrytown USA). The serum insulin levels were studied by electrochemiluminescent immunoassay method (Elecsys 2010, Cobas, Roche Diagnostic, Mannheim, Germany). Insulin resistance was qualified by the homeostasis model assessment formula (14).

Thyroid stimulating hormone (TSH) and free T4 (fT4) levels were quantified via chemiluminescent microparticle immunoassay (Abbott, Architect i2000, Abbott Laboratories Diagnosis Division, IL, USA). Chemiluminescent competitive immunoassay (Advia Centaur XP, Siemens, Tarrytown, USA) was used for the measurement of anti-thyroglobulin antibody (anti-TG Ab) and anti-TPO Ab levels. Reference range was as follows for each: TSH: 0.35-4.94 *µ*IU/mL, fT4: 0.7-1.48 ng/dL, anti-TG: 0-60 U/mL, anti-TPO: 0-57 U/mL. Levels above the upper limits of anti-TPO Ab and anti-TG Ab were considered as positive.

Carotid intima-media thickness (CIMT) was estimated by the noninvasive high-resolution ultrasound of the common carotid arteries (Hitachi, Japan; EUB 7000) with 13 MHz linear probe. The carotid intima-media thickness was defined as the distance between the blood-intima and media-adventitia boundaries and the mean value of consecutive three measurements was taken baseline for CIMT. Measurements were carried out from the localization of 1-centimeter distance after the internal carotid arterial bifurcation, where the hemodinamia had been affected minimum, on B-mode imaging. The same researcher performed all measurements.

### Statistical analysis

The statistical analysis was carried out with the SPSS statistical software (version 18; SPSS, Chicago, IL, USA). Kolmogorov-Smirnov analysis was done to access normality of the variables. Sample t-tests and Mann-Whitney U test was used for the comparison of two group’s distributed variables. Continuous variables were tested by Pearson correlation coefficient and Spearman’s rho correlation coefficient test was done to assess the non-normally distributed variables. P values of <0.05 were determined statistically significant.

## Results

One hundred eighty four patients with PCOS and 106 controls were recruited in the study. Mean age was 23.9±5.6 for PCOS group and 24.3±4.3 for controls, (p>0.05). BMI, waist-hip ratio, fasting blood glucose, fasting insulin, HOMA-IR, triglyceride (TG), low-density lipoprotein cholesterol (LDL-C) and CIMT were higher in PCOS patients (p<0.001, p<0.001, p<0.001, p<0.001, p<0.001, p<0.001, p<0.001, p<0.01 respectively). We didn’t find a significant difference in total cholesterol (TC) levels between two groups (p>0.05), high-density lipoprotein cholesterol (HDL-C) was found lower in PCOS group significantly (p<0.001). Principal data of two groups were represented in Table 1. 

We did not determine a statically significant difference in TSH and fT4 between the groups (p>0.05). We defined that anti-TPO Ab and anti-TG Ab levels were higher in PCOS group in contrast with controls significantly (p<0.001). Thyroid function tests belong to two groups were demonstrated in Table 2.

Anti-TPO Ab was positive in 55 (37.9%) subjects of PCOS group and 11 (11.1%) subjects of controls (Table 3). As also, subjects with positive anti-TG Ab were 22 (15.3%) in PCOS group and 5 (5.1%) in controls (Table 3). Odd’s ratio was calculated as 4.88 for anti-TPO Ab positivity (CI 95%: 2.40-9.95) and 3.39 for anti-TG Ab positivity (CI 95%: 1.24-9.28) (Table 3). Anti-TPO Ab and anti-TG Ab positivity prevalence were determined significantly higher in PCOS patients (respectively; p<0.001, p=0.013) (Table 3). We did not ascertain any correlation between thyroid autoantibody levels and BMI, waist-hip ratio, CIMT and other biochemical parameters. CIMT had a positive correlation with BMI (p<0.001; r=0.350), waist-hip ratio (p=0.023; r=0.194), HOMA-IR (p<0.001; r=0.310) and a negative correlation with HDL-C levels (p<0.01; r=-0.215) (Table 4).

## Discussion

PCOS is the most common reason for medically treatable anovulatory dysfunction (10). Therefore, accurate diagnosis, treatment, and follow-up are substantially important in this patient group. Thyroid function tests are one of the primary studies in the evaluation of menstrual dysfunctions and concurrent thyroid abnormalities for the correct diagnosis of PCOS.

Calvar et al. (12) represented that autoimmune thyroiditis and subclinical hypothyroidism are five times higher in PCOS group than controls. Different studies demonstrated that autoimmune thyroiditis, subclinical and clinical hypothyroidism are associated with PCOS and they recommended to evaluate thyroid function tests periodically in this patient group (11,15). Our study revealed that thyroid autoantibodies are highly positive in patients with PCOS although normal thyroid hormone levels. Calvar et al. (12) established a positive correlation between thyroid dysfunctions and HOMA-IR. In our study, there was no correlation between thyroid autoantibody levels and other parameters.

Du and Li (16) demonstrated a meta-analysis of 6 studies including 726 PCOS patients and 879 controls to evaluate the relationship between PCOS and thyroid autoimmunity. The results showed that autoimmune thyroid disease and thyroid autoantibody levels are higher in PCOS and they conceived that PCOS can be a disorder based on an autoimmune background (16).

Obesity is a metabolic disorder associated with PCOS with the prevalence of 35-70% (17). Many studies concluded that PCOS patients have higher fasting plasma glucose, HOMA-IR, LDL-C, TG and lower HDL-C levels (5,6,18,19). We determined that BMI, waist-hip ratio, fasting blood glucose, fasting insulin, HOMA-IR, TG and LDL-C levels are higher; HDL-C level was lower significantly in PCOS group as against to controls. Carotid intima-media thickness is an important identifier for premature atherosclerosis and different studies confirmed that CIMT is significantly higher in PCOS patients who have an increased risk for cardiovascular morbidities (20,21). In our study, CIMT was statically significant higher in PCOS patients and had a positive correlation with BMI, waist/hip ratio, and HOMA-IR similarly.

Infertility is a difficult issue in PCOS patients. Thyroid autoimmunity is associated with infertility, miscarriage, probable thyroid disorders during pregnancy and in the postpartum period (7). These disorders may also cause complications as gestational hypertension, preeclampsia, pre-term delivery, postpartum haemorrhage and lower birth weight (9). Poppe et al. (8) reported that TSH and anti-TPO antibodies are significantly higher in infertile patients with different reasons (endometriosis, tubal and ovarian pathologies). Another study demonstrated that autoimmune thyroid disease prevalence in infertile women is 16% and statically significantly higher than controls (22). Bellver et al. (23) represented that autoimmune thyroid disease is higher in PCOS patients and have a strong correlation with unexplained infertility and implantation failure (23). Ott et al. (24) also pointed out the relationship between PCOS patients with higher anti-TPO levels and insufficient therapy response in infertile patients who administered clomiphene citrate and metformin. 

In conclusion; we showed that thyroid autoantibody positivity prevalence of euthyroid PCOS patients is 3.5 times higher with respect to control group. Although thyroid hormone level evaluation is fundamental for PCOS follow-up and treatment, thyroid autoantibody assessment is mostly neglected. From all these close relations with PCOS and thyroid dysfunctions, we suggest evaluating both thyroid autoantibodies and hormone levels in PCOS patients at the initial visit and euthyroid patients with positive results for autoantibodies should be followed up closely for the possible thyroid disorders and relevant complications.

## Figures and Tables

**Table 1:**
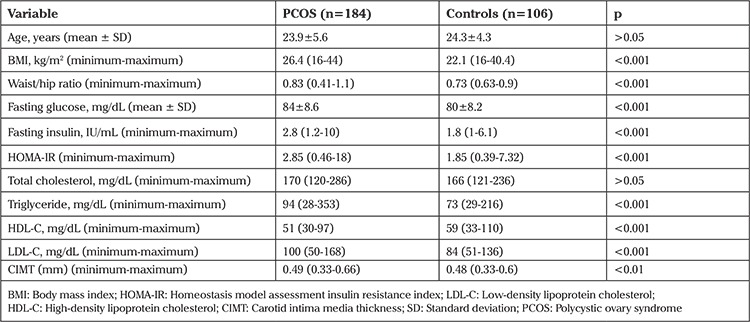
The clinical and biochemical data of patients with PCOS and controls

**Table 2:**
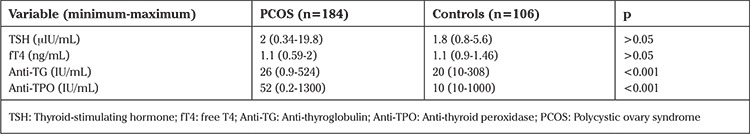
Thyroid function test results of groups

**Table 3:**
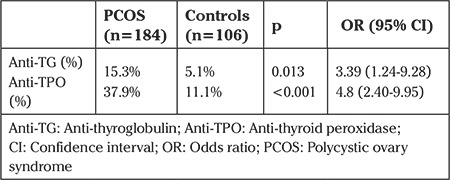
Thyroid autoantibody positivity and odds ratio for PCOS and control groups

**Table 4:**
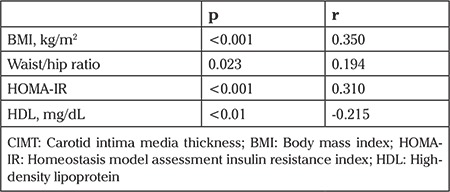
Parameters and their correlations with CIMT
